# Boundary layer transition modeling on leading edge inflatable kite airfoils

**DOI:** 10.1002/we.2329

**Published:** 2019-03-26

**Authors:** Mikko Folkersma, Roland Schmehl, Axelle Viré

**Affiliations:** ^1^ Wind Energy Section, Faculty of Aerospace Engineering Delft University of Technology Delft The Netherlands

**Keywords:** kite airfoils, RANS, sailwings, transition modeling

## Abstract

We present a computational fluid dynamic analysis of boundary layer transition on leading edge inflatable kite airfoils used for airborne wind energy generation. Because of the operation in pumping cycles, the airfoil is generally subject to a wide range of Reynolds numbers. The analysis is based on the combination of the shear stress transport turbulence model with the $\gamma -\tilde{R}e_{\theta t}$ transition model, which can handle the laminar boundary layer and its transition to turbulence. The implementation of both models in OpenFOAM is described. We show a validation of the method for a sailwing (ie, a wing with a membrane) airfoil and an application to a leading edge inflatable kite airfoil. For the sailwing airfoil, the results computed with transition model agree well with the existing low Reynolds number experiment over the whole range of angles of attack. For the leading edge inflatable kite airfoil, the transition modeling has both favorable and unfavorable effects on the aerodynamics. On the one hand, the aerodynamics suffer from the laminar separation. But, on the other hand, the laminar boundary layer thickens slower than the turbulent counterpart, which, in combination with transition, delays the separation. The results also indicate that the aerodynamics of the kite airfoil could be improved by delaying the boundary layer transition during the traction phase and tripping the transition in the retraction phase.

## 1 INTRODUCTION

Airborne wind energy (AWE) is a novel renewable energy technology that uses tethered flying devices to harness wind energy at minimal cost of materials. The devices operate at higher altitudes than conventional wind turbines where the wind is generally stronger and steadier.(1, 2) Consequently, the availability of power and the capacity factor are expected to increase significantly compared with the conventional wind turbines. Also, the cost of the system is expected to decrease dramatically since less materials are used due to the absence of the massive support structure (tower and long blades) of the wind turbine.(3, 4) However, the technology comes also with many challenges. One of these is related to the large traction forces in the tether connecting the flying device to the ground. This adds overall complexity and requires a sophisticated controller to keep the flying device airborne. The tether also adds significant drag to the system. In fact, this drag contribution may be dominant compared with the drag of the wing, depending on the tether length and the size of the system.^5^ This is a major difference when designing an AWE device compared with a conventional aircraft. The conventional gliding (lift‐to‐drag) ratio of the wing alone is not a good measure for the efficiency of the kite. A high lift is more desirable than a low drag, and therefore, high‐lift devices are used as wing design. Thus, in the context of the AWE, an accurate aerodynamic model is crucial to design feasible systems that are both aerodynamically efficient and of high steering capability. For further background information about the AWE technology, the reader is referred to literature.(6, 7, 8, 9)

**Figure 1:**
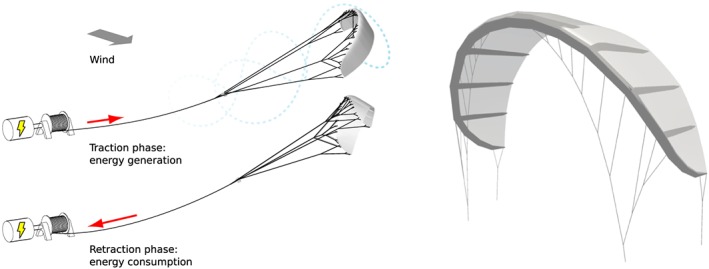
Pumping cycle based airborne wind energy (AWE) system components (left)^10^ and leading edge inflatable (LEI) kite (right) [Colour figure can be viewed at wileyonlinelibrary.com]

Most of the current AWE systems use tethered wings to harness the energy. These kites fly fast crosswind motion, which increases the apparent wind velocity and therefore the aerodynamic efficiency. In this paper, the focus is on traction power kites, which translate the aerodynamic forces from the wind to traction forces in the tether. As illustrated in Figure 1 (left), the kite is operated in pumping cycles, each distinguishing two phases, during which the tether is reeled out and reeled in. During the traction phase, the kite is flown in fast crosswind maneuvers and the generated high traction force is used to pull the tether from a drum, which drives a ground‐based generator. The retraction phase begins when a certain tether length or altitude is reached. By decreasing the angle of attack of the wing, the tension force in the line is minimized and the drum‐generator unit is used to pull the kite towards the ground, using a relatively small amount of the energy generated in the traction phase. Thus, the kite experiences a wide range of different conditions during the cyclic operation. During the traction phase, the apparent wind velocity and the Reynolds number are high, and the angle of attack is close to the critical stall angle. During the retraction phase, the kite is not flying crosswind motion, the Reynolds number and the angle of attack are rather low to minimize the lift force.(10, 11)

This paper focuses on leading edge inflatable (LEI) kites. As depicted in Figure 1, this type of kite consist of a fabric membrane, a tubular inflatable structure, and a bridle line system. The aerodynamic load acting on the canopy is collected by the tubular frame and transferred to the bridle line system. The structure is very lightweight and flexible, and therefore, its shape is changing with the varying loading. Thus, the distributed aerodynamic load and the structural deformation are strongly coupled and define a fluid‐structure interaction (FSI) problem. The kite has a low aspect ratio and a high anhedral angle, which results in significant three‐dimensional flows such as spanwise crossflow and wingtip vortices. Former studies on LEI kite aerodynamics include numerical models by Breukels^12^ and Bosch,^13^ which are based on two‐dimensional section data computed with computational fluid dynamics (CFD). Such types of models are fast, but they have not been properly validated, and they do not incorporate the essential three‐dimensional flow components. Wind tunnel experiments, on the other hand, are challenging because a large inflatable membrane structure can not be scaled down adequately, to fit into the tunnel test section. Consequently, experimental studies have generally aimed at in situ aerodynamic characterization during field tests, measuring global properties such as line tension, apparent wind velocity, and angle of attack.(14, 15)

**Figure 2:**
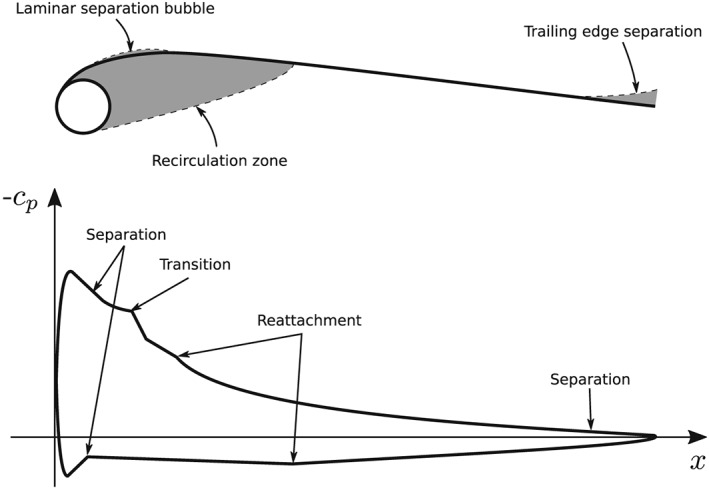
Flow topology around the leading edge inflatable (LEI) kite airfoil

In this work, the focus is on two‐dimensional kite airfoil aerodynamics. As illustrated in Figure 2 (top), the airfoil of the LEI kite consists of a tube and a curved thin canopy. Commonly, these airfoils with circular leading edge and membranes are called sailwings. The sailwing airfoils have been widely studied historically in the context of sailing. More recently, they have also been used as a lightweight and cheap wing design in several applications such as airplanes,^16^ hang gliders,^17^ and wind turbine blades.^18^ In those applications, the sail is attached to a frame made of stiff material, such as carbon fiber or aluminum, and the joint on the suction side between the circular leading edge and the membrane is smooth. These single membrane sailwing sections have been investigated experimentally by several authors towards the end of the 1970s and in the beginning of the 1980s.(19, 20, 21) However, the studies are mostly quantitative and use rather low Reynolds numbers. Defining the Reynolds number with the chord length *c* of the airfoil, the range of variation is typically between Re = 10^5^ and 7.5 × 10^5^, which is only representative of the retraction phase of AWE systems. During the traction phase, the Reynolds numbers encountered by the kite are in the order of from 10^6^ to 10^8^. There is thus a need to extend the range of the Reynolds numbers to values of relevance to the traction phase.

The flow topology around the airfoil and the corresponding pressure coefficient c_p_ are shown in Figure 2. The flow on the suction side is similar to a conventional airfoil, but on the pressure side, a recirculation zone is formed behind the leading edge tube. The size of the zone depends on the angle of attack and the Reynolds number. Furthermore, it is yet unclear what the impact of boundary layer transition is on the aerodynamic performance. Given the range of the Reynolds numbers of interest for AWE and the specific topology of the LEI kite airfoil, the flow separation on the suction side may occur already from a laminar boundary layer, which may lead to a leading edge stall at rather low angles of attack. It has been shown in the literature that, at low angles of attack, the lift force may change dramatically depending on whether the separation occurs from a laminar or a turbulent boundary layer.^22^ Furthermore, it is well known that a cylinder encounters a drag crisis in which the drag coefficient decreases suddenly when the Reynolds number increases. Since the LEI kite airfoil has a circular leading edge, the boundary layer transition may have a significant effect on the size of the recirculation zone behind the circular leading edge. The chord‐length‐to‐leading‐edge‐diameter ratio defines the ratio of the two Reynolds numbers with different characteristic lengths. In the present work, the ratio is approximately 10 for the LEI kite airfoil, and therefore, the diameter Reynolds numbers are one magnitude lower than the chord Reynolds numbers. The drag crisis occurs for a cylinder at around Re_D_ = 3 × 10^5^, and therefore, for the present airfoil, the drag crisis is expected in the range of Re = 3 × 10^6^, which is in the lower end of the traction phase and in the higher end of the retraction phase. The boundary layer transition is usually neglected in conventional turbulence models used in CFD. Only recently, generic transition models have been developed within the framework of Reynolds averaged Navier‐Stokes (RANS) on arbitrary unstructured meshes.(23, 24)

A flexible airfoil with a membrane adapts itself with the flow. Boer^21^ shows that the maximum camber of the sailwing airfoil moves towards the leading edge when the angle of attack is increased, which both extends the practical angles of attacks and improves the maximum gliding ratio in comparison to similar rigid airfoil. Also, the maximum gliding ratio occurs with high lift coefficient, which is beneficial for the AWE systems with the additional tether drag.^19^ The disadvantages of the flexible airfoil are the increased complexity of the model due to the FSI coupling and the elongation and the durability of the membranes.

In this work, the shear stress transport (SST) turbulence model^25^ is used with and without the $\gamma -\tilde{R}e_{\theta t}$ transition model^23^ to find out the significance of the transition modeling. The simulations are carried out for a full range of Reynolds numbers, in order to identify the important flow effects that impact on the aerodynamics of the LEI kite airfoil. For simplicity, the airfoil is assumed to be rigid. The paper is organized as follows. Section 2 introduces the CFD solver and focuses especially to the turbulence and transition models. In Section 3, we address the largest numerical uncertainty in CFD simulations, which is caused by the mesh, then we carry out a validation assessment by comparing the numerical results with experimental results. Subsequently, we present the main results from the simulations around the LEI kite airfoil. Finally, we conclude the paper in Section 4.

## 2 COMPUTATIONAL MODELING

The simulations are based on the OpenFOAM v1706 toolbox developed by ESI.^26^ OpenFOAM is an open‐source CFD library written in C++ and has a high‐level programming interface to solve partial differential equations. The Navier‐Stokes equations are discretized on a polyhedral mesh using a cell‐centered finite volume method. Several solvers and turbulence models are prepackaged in the default distribution. Because we consider a low Mach number flow, the fluid is assumed incompressible and the simulations are accordingly performed with the incompressible and steady‐state solver simpleFoam.

### 2.1 Turbulence model

One of the main uncertainties in CFD and aerodynamics is the turbulence model, which is practically required for flows at high Reynolds number. The most common approach are the RANS equations, in which the transport equations are solved only for the mean flow field and the turbulent fluctuations are represented by model terms. The RANS equations for the steady‐state incompressible fluid are given by

(1) $$\frac{\partial U_{i}}{\partial x_{i}}=0,$$

(2) $$U_{j}\frac{\partial U_{i}}{\partial x_{j}}=-\frac{1}{\rho }\frac{\partial P}{\partial x_{i}}+\nu \frac{\partial^{2}U_{i}}{\partial x_{j}^{2}}x-\frac{\partial \bar{u_{i}^{\prime }u_{j}^{\prime }}}{\partial x_{j}},$$

where U_i_ and P are the mean velocity and pressure, respectively, and $\bar{u_{i}^{\prime }u_{j}^{\prime }}$ is the Reynolds stress term which represents the effect of the turbulent velocity fluctuations and which has to be modeled. Many RANS turbulence models have been developed for different applications but none of them works universally for all types of flows. Thus, it is crucial to critically assess the suitability of a specific model for each application case.

The most widely used RANS‐based turbulence models employ two transport equations for turbulence properties, generally one for the turbulence kinetic energy k and another one for the turbulence length or timescale. Popular for engineering applications is the SST model by Menter,^25^ which combines the k − ϵ model by Jones and Launder^27^ and the k − ω model by Wilcox^28^ to get the best of both modeling concepts. OpenFOAM v1706 uses a revised version of the SST model^29^ with the turbulence specific dissipation rate production term formulated by Menter and Esch.^30^ The term is limited similarly as in the transport equation for the turbulence kinetic energy described by on the NASA webpages.^31^ The transport equation for the turbulence kinetic energy is given by

(3) $$\frac{\partial k}{\partial t}+\frac{\partial U_{j}k}{\partial x_{j}}={\tilde{P}}_{k}-\beta^{*}k\omega +\frac{\partial }{\partial x_{j}}\left[(\nu +\sigma_{k}\nu_{t})\frac{\partial k}{\partial x_{j}}\right],$$

where ${\tilde{P}}_{k}$ is the limited production term

(4) $${\tilde{P}}_{k}=\min(P_{k},10\beta^{*}k\omega ),$$

with

(5) $$P_{k}=\nu_{t}\frac{\partial U_{i}}{\partial x_{j}}\left(\frac{\partial U_{i}}{\partial x_{j}}+\frac{\partial U_{j}}{\partial x_{i}}\right).$$

The transport equation for the specific turbulence dissipation rate ω is given by

(6) $$\frac{\partial \omega }{\partial t}+\frac{\partial U_{j}\omega }{\partial x_{j}}=\gamma \frac{{\tilde{P}}_{k}}{\nu_{t}}-\beta \omega^{2}+\frac{\partial }{\partial x_{j}}\left[(\nu +\sigma_{\omega }\nu_{t})\frac{\omega }{x_{j}}\right]+2(1-F_{1})\sigma_{\omega 2}\frac{1}{\omega }\frac{\partial k}{\partial x_{j}}\frac{\partial \omega }{\partial x_{j}},$$

where F_1_ is the blending function that switches between the two elementary turbulence models

(7) $$F_{1}=\tanh\left[{\left[\min\left[\max\left(\frac{\sqrt{k}}{B^{*}\omega y},\frac{500\nu }{y^{2}\omega }\right),\frac{4\sigma_{\omega 2}k}{CD_{k\omega }y^{2}}\right]\right]}^{4}\right],$$

with

(8) $$CD_{k\omega }=\max\left(2\sigma_{\omega 2}\frac{1}{\omega }\frac{\partial k}{\partial x_{j}}\frac{\partial \omega }{\partial x_{j}},10^{-10}\right).$$

The blending function F_1_ depends on the distance y from the surface. Far from the surface, F_1_ is zero, and the k − ϵ model is used. Inside the boundary layer, F_1_ is close to unity, and the k − ω model is used. The constants are also interpolated as follows:

(9) $$\phi ={F{}_{1}\phi }_{1}+{(1-F_{1})\phi }_{2},$$

and they are given by

(10) $$\begin{array}{ccc} \alpha_{1} & =5/9,\beta_{1}=3/40,\gamma_{1}=5/9,\sigma_{k1}=0.85,\sigma_{\omega 1}=0.5, & \\ \alpha_{2} & =0.44,\beta_{2}=0.0828,\gamma_{2}=0.44,\sigma_{k2}=1,\sigma_{\omega 2}=0.856, \end{array}$$

and β ^∗^ = 0.09. The turbulence eddy viscosity is computed from

(11) $$\nu_{t}=\frac{a_{1}k}{\max(a_{1}\omega ,SF_{2})},$$

where S is the magnitude of the strain rate tensor, a_1_ = 0.31, and F_2_ is the blending function

(12) $$F_{2}=\tanh\left[{\left[\max\left(\frac{2\sqrt{k}}{\beta^{*}\omega y},\frac{500\nu }{y^{2}\omega }\right)\right]}^{2}\right].$$

Common turbulence models such as SST cannot describe transition from laminar to turbulent flow and consequently assume that boundary layers are always turbulent. A similar effect can be achieved experimentally by tripping the boundary layer from the very beginning, which leads to a nearly immediate transition to the turbulent boundary layer. Also, in some aerodynamic flows, the transition may be neglected when the Reynolds number is very high and the transition occurs almost immediately when the flow approaches the surface. However, the laminar boundary layer and the transition may have a significant effect when the Reynolds number is not very high.

### 2.2 Transition model

Boundary layer transition is a challenging topic that has been extensively studied. Three primary modes of laminar‐turbulent transition are considered^32^

1. Natural transition: Perturbations from the exterior flow, such as turbulence propagate into the boundary layer. The perturbations grow and through Tollmien‐Schlichting waves and turbulent spots, eventually the boundary layer becomes turbulent. In external aerodynamic flows around smooth surfaces, this is the usual mode of transition.
2. Bypass transition: Either the freestream disturbances are high (turbulence intensity) and propagate directly into the boundary layer or the surface is rough. Consequently, the turbulence is directly transported to the boundary layer which triggers transition. For example, the deforming membrane or the woven material of the kite may induce disturbances which trigger transition.
3. Separation induced transition: A laminar boundary layer cannot withstand much adverse pressure gradient, and therefore, it separates rather easily. Flow separation induces disturbances and transitions quickly. The turbulence enhances mixing, and therefore, the formed shear layer may reattach again. The size of these separation bubbles varies heavily and long laminar separation bubbles may affect the flow significantly. The laminar separation bubbles are observed around the airfoils with rather low Reynolds numbers.

For a more comprehensive review of the boundary layer transition, the reader is referred to literature.(33, 34)

The presence of different modes is one reason for the complexity of transition, especially within the RANS framework, because the transport equations are only resolved for the mean flow field and do therefore not contain the intrinsic information of the disturbances. Moreover, common transition models rely on integral boundary layer values to predict transition, which are costly to compute on arbitrary unstructured meshes. Transition also does not have a clear definition in three dimensions. Therefore, general purpose transition models for RANS simulations have not been available until recently. Langtry and Menter^23^ developed the $\gamma -\tilde{R}e_{\theta t}$ transition model on top of the SST turbulence model, but it has also been coupled with other turbulence models such as Spalart‐Allmaras.^35^ The transition model does not try to capture the actual physics of the transition but is based on empirical correlations. It has been successfully employed for several external aerodynamic problems such as flow around airfoils and turbine blades^32^ and cylinder.^36^

Two additional transport equations are used to model transition. The transported quantities are intermittency γ and transition onset momentum thickness Reynolds number $\tilde{R}e_{\theta t}$. The intermittency controls the transition by scaling the production and destruction terms of the turbulence kinetic energy k. The Reynolds number $\tilde{R}e_{\theta t}$ quantifies the effect of the freestream conditions to inside the boundary layer. The transport equation for the intermittency is given by

(13) $$\frac{\partial \gamma }{\partial t}+\frac{\partial U_{j}\gamma }{\partial x_{j}}=P_{\gamma }-E_{\gamma }+\frac{\partial }{\partial x_{j}}\left[\left(\nu +\frac{\nu_{t}}{\sigma_{f}}\right)\frac{\partial \gamma }{\partial x_{j}}\right],$$

where P_γ_ and E_γ_ are the production and destruction source terms. The production term is defined as

(14) $$P_{\gamma }=F_{\text{length}}c_{a1}S\sqrt{\gamma F_{\text{onset}}}(1-c_{e1}\gamma ),$$

with

(15) $$F_{\text{onset}}=\max\left(F_{\text{onset2}}-F_{\text{onset3}},0\right),$$

(16) $$F_{\text{onset1}}=\frac{{\text{Re}}_{v}}{2.193{\text{Re}}_{\theta c}},$$

(17) $$F_{\text{onset2}}=\min\left(\max\left(F_{\text{onset1}},F_{\text{onset1}}^{4}\right),2.0\right),$$

(18) $$F_{\text{onset3}}=\max\left(1-{\left(\frac{R_{T}}{2.5}\right)}^{3},0\right),$$

(19) $${\text{Re}}_{v}=\frac{y^{2}S}{\nu },\;R_{T}=\frac{k}{\nu \omega },$$

where F_length_ is the empirical correlation that controls the transition length. The transition onset is controlled by F_onset_, which is triggered when the vorticity Reynolds number Re_v_ sufficiently exceeds the critical momentum thickness Reynolds number Re_θc_, which is also represented by an empirical correlation. Both correlations are functions of the transported Reynolds number $\tilde{R}e_{\theta t}$ and defined in Langtry and Menter.^23^

The destruction term is defined by

(20) $$E_{\gamma }=c_{a2}\Omega \gamma F_{\text{turb}}(c_{e2}\gamma -1),$$

where Ω is the vorticity magnitude, and F_turb_ is the function that disables the destruction term outside of laminar boundary layer or in the viscous sublayer

(21) $$F_{\text{turb}}=e^{-{\left(\frac{R_{T}}{4}\right)}^{4}}.$$

The intermittency is modified to take the separation induced transition into account by defining

(22) $$\gamma_{\text{eff}}=\max(\gamma ,\gamma_{\text{sep}}),$$

(23) $$\gamma_{\text{sep}}=\min\left(2\max\left[0,\left(\frac{{\text{Re}}_{v}}{3.235{\text{Re}}_{\theta c}}\right)-1\right]F_{\text{reattach}},2\right)F_{\theta t},$$

(24) $$F_{\text{reattach}}=e^{-{\left(\frac{R_{T}}{20}\right)}^{4}}.$$

The transport equation for the transition onset Reynolds number $\tilde{R}e_{\theta t}$ is given by

(25) $$\frac{\partial \tilde{R}e_{\theta t}}{\partial t}+\frac{\partial U_{j}\tilde{R}e_{\theta t}}{\partial x_{j}}=P_{\theta t}+\frac{\partial }{\partial x_{j}}\left[\sigma_{\theta t}(\nu +\nu_{t})\frac{\partial \tilde{R}e_{\theta t}}{\partial x_{j}}\right],$$

where P_θt_ is the source term, which forces the $\tilde{R}e_{\theta t}$ to match the local empirical correlation Re_θt_ value outside the boundary layer. The source term is given by

(26) $$P_{\theta t}=\frac{c_{\theta t}}{t}({\text{Re}}_{\theta t}-\tilde{R}e_{\theta t})(1.0-F_{\theta t}),$$

where t = 500ν/U ^2^ is the timescale, and U is the velocity magnitude. The blending function F_θt_ turns on the source term outside the boundary layer, which diffuses $\tilde{R}e_{\theta t}$ inside the boundary layer

(27) $$F_{\theta t}=\min\left(\max\left(F_{wake}e^{{\left(-\frac{y}{\delta }\right)}^{4}},1.0-{\left(\frac{\gamma -1/c_{e2}}{1.0-1/c_{e2}}\right)}^{2}\right),1.0\right),$$

(28) $$F_{\text{wake}}=e^{-{\left(\frac{{\text{Re}}_{\omega }}{10^{5}}\right)}^{2}};\;{\text{Re}}_{\omega }=\frac{\omega y^{2}}{\nu },$$

(29) $$\delta =\frac{375\Omega y\nu \tilde{R}e_{\theta t}}{U^{2}},$$

where F_wake_ is the function, which turns off the blending function in the wake regions. The constants in the transport equations are defined as

(30) $$\begin{array}{ccccccccc} c_{a1} & =2.0, & c_{a2} & =0.06, & c_{e1} & =1.0, & c_{e2} & =50, & \\ \sigma_{f} & =1.0, & \sigma_{\theta t} & =2.0, & c_{\theta t} & =0.03. \end{array}$$

The transition model is coupled with the SST turbulence model by scaling the turbulence kinetic energy production and destruction terms by intermittency as

(31) $${\tilde{P}}_{k}=\gamma_{\text{eff}}{\tilde{P}}_{k},$$

(32) $$D_{k}=\min(\max(\gamma_{\text{eff}},0.1),1.0)D_{k},$$

and the blending term as

(33) $$F_{1}=\max(F_{1},F_{3}),\;F_{3}=e^{-{\left(\frac{R_{y}}{120}\right)}^{8}},\;R_{y}=\frac{y\sqrt{k}}{\nu }.$$

### 2.3 Boundary conditions

For the far field, a mixed boundary condition is applied depending on whether the flow enters or leaves the computational domain. For inflow, uniform values are imposed for the velocity and the turbulence quantities, while the pressure gradient is set to zero. For outflow, a uniform pressure is imposed, while the gradients of velocity and turbulence quantities are set to zero. At the airfoil surface, a no‐slip boundary condition is applied. The inlet values for the turbulence quantities are calculated from the turbulence intensity and the eddy viscosity ratio. Spalart and Rumsey^37^ recommend using following formula for the eddy viscosity at the inlet boundary condition

(34) $$\nu_{t}/\nu =2\times 10^{-7}\text{Re}.$$

The sensitivity of the results to the eddy viscosity ratio is assessed on the LEI kite airfoil at Re = 10^6^ for α = 0° and α = 10°. The eddy viscosity ratio is varied from 0.1 to 10, and the results do not change significantly, and therefore, ν_t_/ν = 10 is used in the following simulations.

The inlet boundary condition for γ is unity, and the other turbulence quantities are estimated as

(35) $$k=\frac{3}{2}{\left(U_{\infty }\frac{Tu}{100}\right)}^{2},$$

with

(36) $$\omega =\frac{k}{\nu }{\left(\frac{\nu_{t}}{\nu }\right)}^{-1},$$

and for $\tilde{R}e_{\theta t}$ with zero acceleration in streamwise direction^23^

(37) $$\begin{array}{ccc} \tilde{R}e_{\theta t} & =1173.51-589.428Tu+\frac{0.2196}{Tu^{2}},\;Tu\leq 1.3, & \\ \tilde{R}e_{\theta t} & =331.5{\left(Tu-0.5658\right)}^{-0.671},\;\;Tu>1.3, \end{array}$$

where Tu is the turbulence intensity in percentages.

OpenFOAM works only with three‐dimensional meshes, and therefore, the mesh has a thickness of one cell in out‐of‐plane direction. An empty boundary condition is applied on the extruded planes to disable the solution calculation in the third dimension.

## 3 RESULTS

We consider two different airfoil geometries. In a first step, we assess the effect of mesh resolution on the results. Subsequently, we validate the numerical model with a sailwing section for which experimental results are available. We conclude with a presentation of the numerical results for the LEI kite airfoil.

### 3.1 Mesh generation and uncertainty assessment

**Figure 3:**
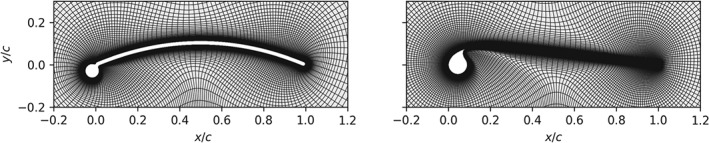
Reference meshes around the sailwing airfoil (left) and the LEI kite airfoil (right) with the mesh resolution of 191 × 144 and 255 × 211, respectively

The computational meshes are generated with the open‐source framework Overture^38^ and illustrated in Figure 3. First, a spline is generated from the airfoil coordinates. The airfoil is slightly modified by filling in the sharp angle at the junction of the circular leading edge and the canopy. This is required for the subsequent extrusion to prevent self‐intersection. Employing an inverse hyperbolic tangent stretching algorithm, the node points are clustered closely to the leading and trailing edges of the airfoil. The spline curve is then extruded in the outwards direction using an hyperbolic extrusion algorithm, keeping a constant geometric ratio. The mesh is extruded approximately 500 chord lengths, and the hyperbolic extrusion tends to form a circular shape for the outer boundary. The resulting mesh is saved in PLOT3D format, which can be directly imported to the OpenFOAM toolbox.

**Figure 4:**
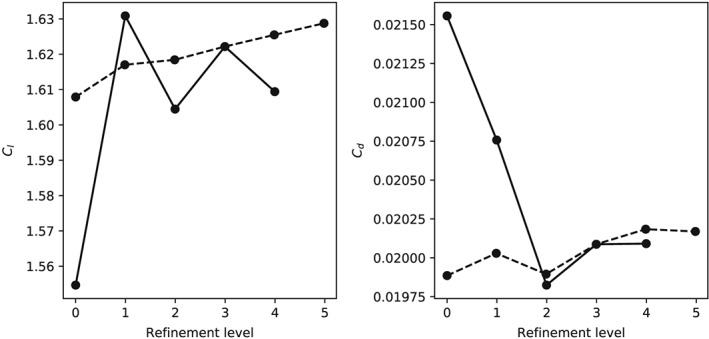
Sensitivity of computed aerodynamic coefficients to mesh refinement. Each refinement level decreases the cell size by a third from the previous level. The solid line is the refinement in the airfoil surface direction, and the dashed line is the refinement in normal direction of the airfoil

To assess the effect of the mesh resolution on the simulation results, we conduct a sensitivity analysis using the $\gamma -\tilde{R}e_{\theta t}$ transition model. The investigated parameters are the number of discretization points along and normal to the airfoil surface. The resolution is increased in each direction consecutively by a factor of 1.5. The computed lift and drag coefficients for increasing resolution along the airfoil surface are given in Table 1 and Figure 4 (left).

**Table 1:** Sensitivity of computed aerodynamic coefficients with respect to mesh resolution along the airfoil surface (Re = 10^6^)^a^

Mesh			*C*_*l*_			*C*_*d*_		
Resolution	Δ*x*_*LE*_/*c*	Δ*x*_*TE*_/*c*	*α* = 0°	*α* = 10°	*α* = 15°	*α* = 0°	*α* = 10°	*α* = 15°
170 × 211	0.00356	0.00242	0.116	1.55	2.01	0.0387	0.0216	0.0323
255 × 211	0.00238	0.00207	0.155	1.63	2.07	0.0396	0.0208	0.0340
383 × 211	0.00158	0.00136	0.172	1.60	2.04	0.0399	0.0198	0.0329
575 × 211	0.00105	0.00103	0.183	1.62	2.06	0.0402	0.0201	0.0334
863 × 211	0.00070	0.00086	0.194	1.61	2.04	0.0403	0.0201	0.0333

a The edge size along the airfoil surface Δ*x* is given at the leading edge (LE) and at the trailing edge (TE).

Three values for the angle of attack are investigated, namely, α = 0°, 10°, and 15°. The mesh dependency of the results decreases for increasing angle of attack. This is probably due to the large recirculation zone on the pressure side that leads to instabilities and therefore to higher mesh sensitivity. Nevertheless, the results are well converged for higher angles of attack (α = 10° and 15°) and satisfactory if the airfoil is aligned with the flow (α = 0°). Thus, the mesh with 575 cells along the surface of the airfoil is adequate. In wall‐normal direction, two different Reynolds numbers are studied to ensure that the first cell size requirement y + <1 is met for high Reynolds numbers. The resulting lift and drag coefficients are given in Table 2 and Figure 4 (right), indicating that 211 points in wall‐normal direction are sufficient for convergence. Therefore, we choose the 575 × 211 mesh for the subsequent computational analysis of the LEI kite airfoil.

**Table 2:** Sensitivity of computed aerodynamic coefficients with respect to mesh resolution normal to the airfoil surface (α = 10°)^a^

Mesh			$y_{\max}^{+}$	$y_{\text{mean}}^{+}$	C_l_		C_d_	
Resolution	**Δy_LE_/c ( × 10^−7^)**	**Δy_TE_/c ( × 10^−7^)**	**Re = 5 × 10^7^**	**Re = 5 × 10^7^**	**Re = 10^6^**	**Re = 5 × 10^7^**	**Re = 10^6^**	**Re = 5 × 10^7^**
575 × 63	4.20	4.11	1.310	0.437	1.61	1.65	0.0199	0.0161
575 × 94	2.65	2.62	0.827	0.277	1.62	1.66	0.0200	0.0159
575 × 141	1.70	1.61	0.524	0.178	1.62	1.66	0.0199	0.0158
575 × 211	1.09	1.13	0.337	0.115	1.62	1.67	0.0201	0.0158
575 × 317	0.729	0.643	0.224	0.077	1.63	1.67	0.0202	0.0159
575 × 475	0.486	0.428	0.149	0.051	1.63	1.67	0.0202	0.0158

a The edge size normal to the airfoil surface Δy is given at the leading edge (LE) and at the trailing edge (TE).

A similar sensitivity analysis is conducted for the sailwing airfoil to be used in the subsequent validation study. The analysis leads to a computational mesh with 287 cells along the airfoil surface and 215 cells normal to the surface.

### 3.2 Validation study with sailwing

**Figure 5:**
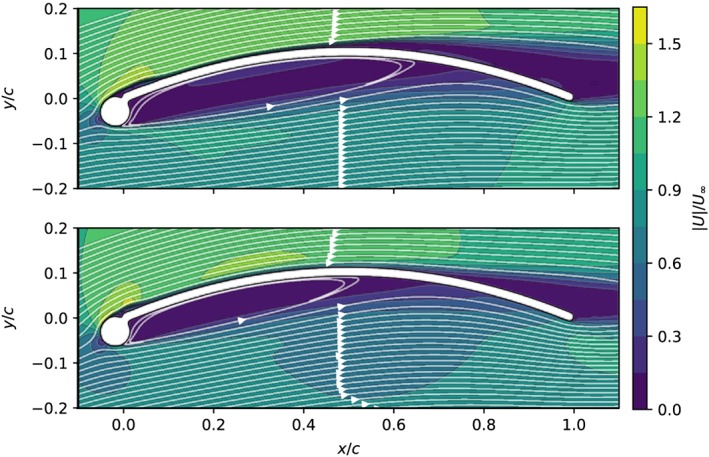
Computed streamlines and normalized flow velocity around the sailwing airfoil, using the transition model, at 7° (top) and 8° (bottom) angle of attack [Colour figure can be viewed at wileyonlinelibrary.com]

Experimental data for LEI kite airfoils is scarce. One of the few available datasets has been published by Bruining^20^ for a sailwing section made of steel consisting of a cylinder and an arc‐shaped plate. The two physical wing elements are not materially joined but only touch each other, forming a small dimple on the suction side of the airfoil. The experiments were carried out in the low speed and low turbulence (LLT) wind tunnel of Delft University of Technology, which has a turbulence intensity of the order of 0.02%. The Reynolds number based on the chord length of the arc plate is Re = 10^5^, and the chord length to the leading edge diameter ratio is c/D = 15. Figure 5 shows the computed flow field for two different values of the angle of attack. The dimple is clearly recognizable on the suction side where arc‐shaped plate and the cylinder are joined.

**Figure 6:**
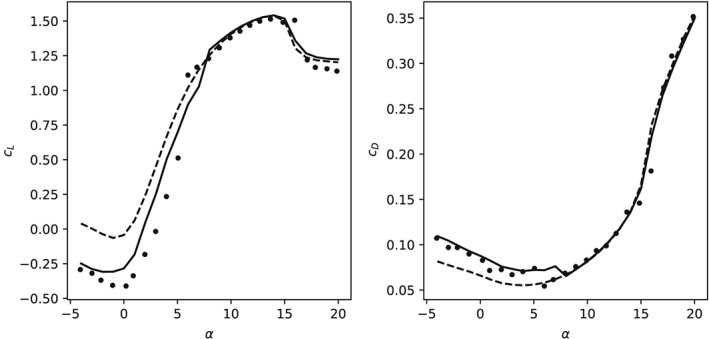
Lift (left) and drag (right) coefficients of the sailwing airfoil as functions of the angle of attack. The experimental results^20^ are shown as marker symbols; the simulation results with transition model are shown as solid line, and the results without transition model as dashed line

**Figure 7:**
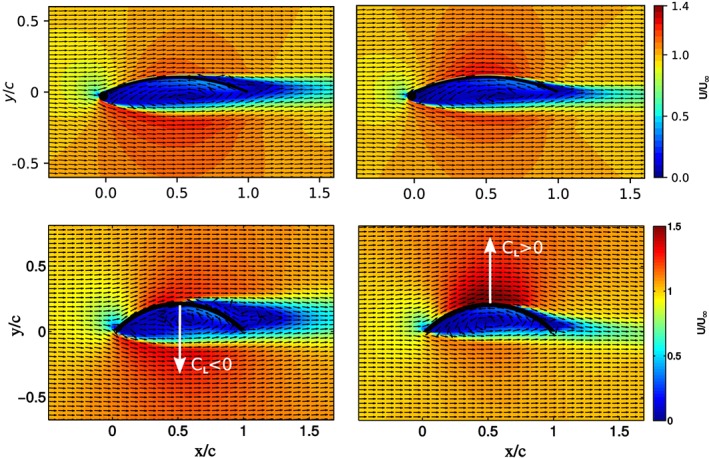
Comparison of the present numerical simulation with the experiment of Bot et al.^22^ Top row: simulation with (left) and without (right) transition model. Bottom row: experiment for Re < 200 000 (left) and Re > 200 000 (right) [Colour figure can be viewed at wileyonlinelibrary.com]

The measured and computed aerodynamic coefficients are shown in Figure 6. We found that the simulations with transition model $\gamma -\tilde{R}e_{\theta t}$ are closer to the experiment than the simulations without transition model. This is expected since the Reynolds number is rather low and the surface of the wind tunnel model is smooth. Furthermore, the numerical results for low angle of attack show a negative lift coefficient when using the transition model and a positive coefficient for fully turbulent boundary layer. This is consistent with the measurements of Bot et al^22^ of the flow around a two‐dimensional curved plate. In Figure 7, we compare these measurements with our sailwing airfoil simulations for zero angle of attack. For Re < 200 000, the measured lift coefficient is negative because the flow separates early from the laminar boundary layer. For Re > 200 000, the boundary layer transitions to turbulence while still fully attached to the airfoil. The intensified turbulent mixing has the well‐known effect of delaying the flow separation. As a result, the measured lift of the plate increases abruptly to a positive value at around Re = 200 000 while the drag decreases. A similar effect can be observed in the numerical simulations of the sailwing airfoil. With transition model, the laminar boundary layer separates from the airfoil, while without transition model the boundary layer is always turbulent, and therefore, the separation is delayed.

From Figure 6, we can see that with increasing angle of attack, the measured aerodynamic coefficients change abruptly at around *α* = 6°. A similarly abrupt change can be identified for the computed coefficients of the sailwing airfoil at *α* = 8°. For higher angles of attack, the boundary layer encounters the adverse pressure gradient increasingly earlier with the effect that also transition is triggered earlier.^39^ Because a turbulent boundary layer can better sustain an adverse pressure gradient, the effect on the aerodynamics is generally positive. Figure 5 shows the computed pressure field and streamlines around the airfoil for *α* = 7° and 8°, which is the angle of attack range in which the lift suddenly increases. The dimple on the suction side of the airfoil, where the two‐wing elements are joined, is occupied by a small separation bubble. At *α* = 0°, this bubble does not disturb the main flow over the airfoil. With increasing angle of attack, the separation bubble enlarges but still reattaches without transition. At *α* = 8°, the transition also occurs within the separation bubble, and therefore, the lift coefficient sharply increases and the drag coefficient decreases. This explains why the simulations with and without transition models lead to very similar results with angles of attack higher than 8°. Overall, the simulations with combination of turbulence and transition models agree well with the experiments, even at large angles of attack.

### 3.3 LEI kite airfoil

The validated simulation setup is used to analyze the LEI kite airfoil for a wide range of Reynolds numbers that are representative for the traction and retraction phases of AWE systems. The airfoil represents the center section of the LEI kite with a chord‐to‐leading‐edge‐diameter ratio of *c*/*D* = 10.8. The angle of attack is varied up to the critical value at which stall occurs. We do not consider configurations beyond this point because the steady‐state solver is not suitable for the inherently unsteady post‐stall flow conditions. The Reynolds number is varied from Re = 10^5^ to 5 × 10^7^. Results are presented for simulations with and without transition modeling.

**Figure 8:**
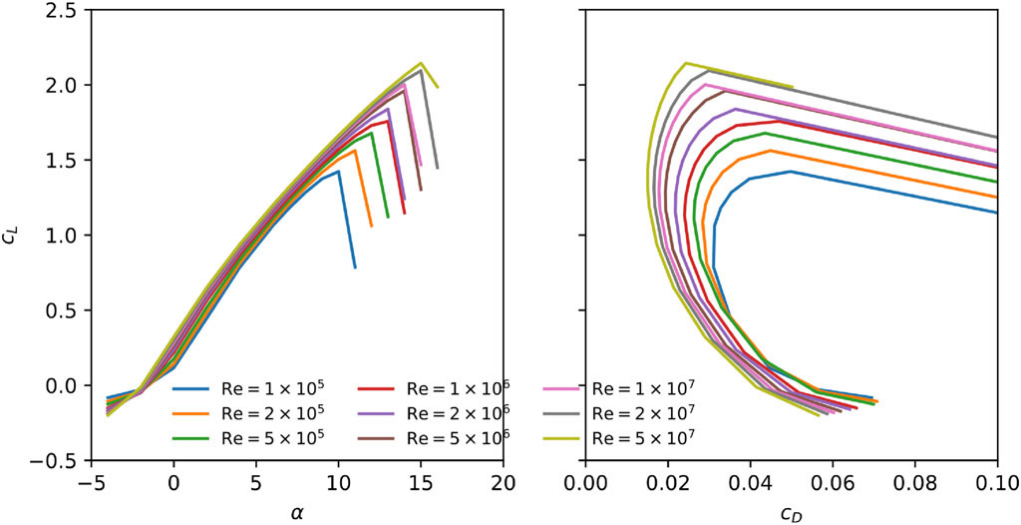
Lift coefficient as function of angle of attack (left) and drag polar (right) for the leading edge inflatable (LEI) kite airfoil at various Reynolds numbers, computed without transition model [Colour figure can be viewed at wileyonlinelibrary.com]

**Figure 9:**
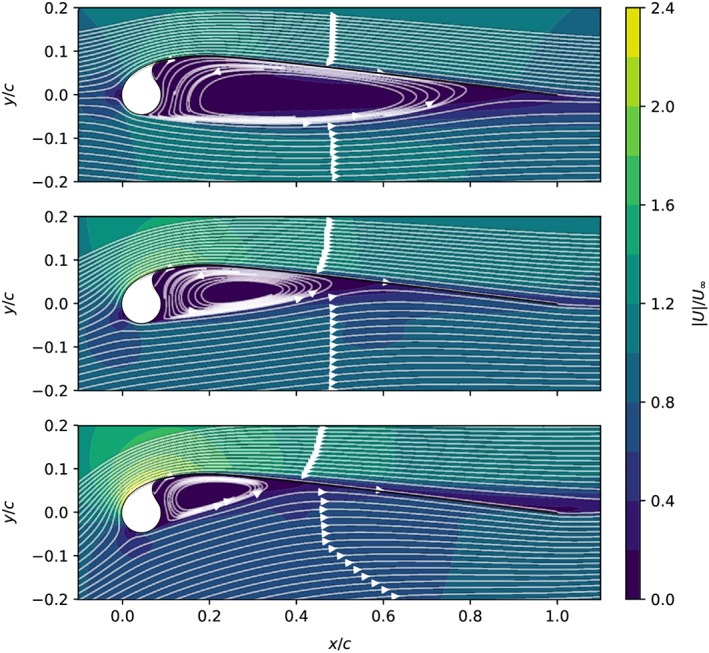
Streamlines and normalized flow velocity around the leading edge inflatable (LEI) kite airfoil, computed without transition modeling at Re = 10^6^ for α = 0° (top), α = 6° (center), and α = 12° (bottom) [Colour figure can be viewed at wileyonlinelibrary.com]

The aerodynamic coefficients computed without transition modeling are illustrated in Figure 8. The general trends are in line with those for conventional airfoils. With increasing Reynolds number, the lift coefficient increases and the drag decreases leading to an improved aerodynamic performance. Also, the critical stall angle increases resulting in higher maximum lift coefficients. Figure 9 illustrates the streamlines and the normalized velocity magnitude around the airfoil at Re = 10^6^ for several values of the angle of attack. As the angle of attack increases, the recirculation zone on the pressure side of the airfoil decreases in size, which increases lift and decreases drag.

**Figure 10:**
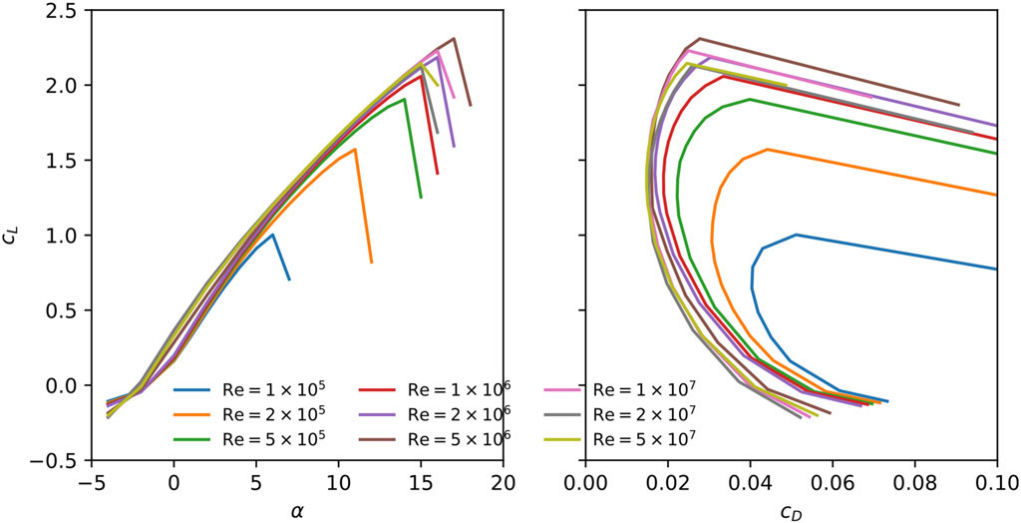
Lift (left) and drag (right) polar curves for the leading edge inflatable (LEI) kite middle section, computed with transition modeling for various Reynolds numbers [Colour figure can be viewed at wileyonlinelibrary.com]

**Figure 11:**
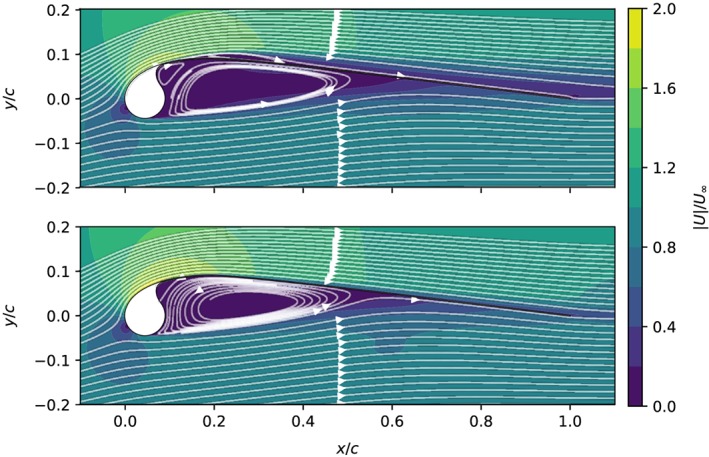
Streamlines and the normalized flow velocity around the LEI kite airfoil, computed with transition modeling at α = 6° for Re =10^5^ (top) and Re =5 × 10^5^ (bottom) [Colour figure can be viewed at wileyonlinelibrary.com]

Taking laminar‐turbulent transition into account significantly affects the flow simulation. Figure 10 shows the aerodynamic coefficients computed with transition model. At the lowest considered Reynolds number, Re = 10^5^, the aerodynamic performance is worse than computed without transition model. The lower lift and higher drag are caused by a long laminar separation bubble on the suction side, as indicated by Figure 11. Although the flow still reattaches, the airfoil stalls at much lower angles of attack, *α* = 6°, because the flow separates relatively early from the laminar boundary layer. Increasing the Reynolds number from Re = 10^5^ to 2 × 10^5^ leads to an increase of the stall angle from 6° to 11° because the boundary layer now transitions to turbulence before the flow separates, which therefore occurs with a delay. Increasing the Reynolds number further, the stall angle increases consistently until reaching the maximum lift coefficient at Re = 5 × 10^6^. Above this value, the stall angle first decreases again, and beyond Re = 2 × 10^7^, the stall angle and maximum lift coefficient begin to increase again.

**Figure 12:**
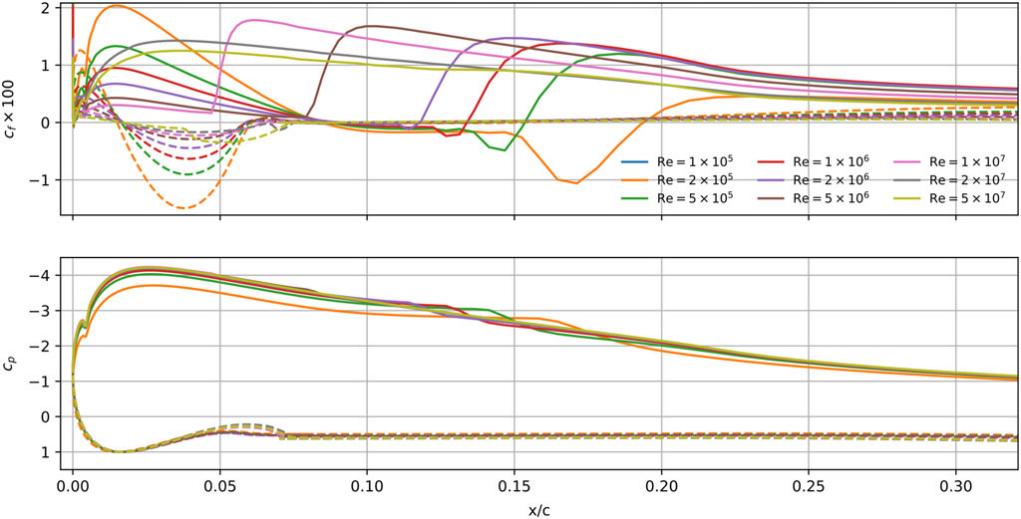
Friction coefficient c_f_ (top) and pressure coefficient c_p_ (bottom) around the sailwing, computed with transition modeling. Solid line is the suction side, and dashed line is the pressure side [Colour figure can be viewed at wileyonlinelibrary.com]

The transition, separation and reattachment regions can be identified on the basis of the skin friction coefficient *c*_*f*_. We use the minimum and maximum value of this coefficient to define the onset and end of the transition region. Figure 12 shows the skin friction and pressure coefficients at *α* = 10° and various Reynolds numbers. At Re = 2 × 10^5^, 5 × 10^5^, 10^6^ and 2 × 10^6^, the negative value of *c*_*f*_ on the suction side indicates a laminar separation bubble. The bubble starts at a roughly constant value *x*/*c* = 0.08; however, the reattachment of the flow varies with the Reynolds number. For Re = 10^5^, 5 × 10^5^, 10^6^ and 2 × 10^6^, we find *x*/*c* = 0.19, *x*/*c* = 0.15, *x*/*c* = 0.13, and *x*/*c* = 0.12, respectively.

At Re = 5 × 10^6^ and above, no laminar separation bubble is observed. Therefore, the flow at Re = 5 × 10^6^ leads to the highest lift coefficient in the range of studied Reynolds numbers. It has the longest, fully attached laminar boundary layer that also remains thinner. Both features increase the lift and decrease the drag. Also, the thin boundary layer can better sustain an adverse pressure gradient, and therefore, the highest stall angle and maximum lift coefficient is observed for Re = 5 × 10^6^. At Re = 5 × 10^7^ and above, the flow separates almost immediately from the leading edge, and therefore, the results are very similar to the simulations without modeling transition. This is consistent with the experiments of Achenbach^40^ for supercritical flow around a cylinder at Re_*D*_ = 1.5 × 10^6^, which is equivalent to Re = 1.6 × 10^7^ for the present sailwing.

At low angles of attack, around *α* = 0°, a sudden jump of lift and drag coefficients is observed when increasing the Reynolds number from Re = 2 × 10^6^ to 5 × 10^6^. This regime corresponds to the critical Reynolds number of the flow around a cylinder, which varies from Re_*D*_ = 2 × 10^5^ to Re_*D*_ = 5 × 10^5^.^40^ In this regime, the boundary layer transitions before the flow separates, and therefore, the flow is longer attached to the airfoil. Consequently, the recirculation zone is slightly smaller, resulting in an increased lift and decreased drag.

To summarize, we can state that during the traction phase with high relative flow velocity and thus high Reynolds number, it is advantageous to increase the stall angle by delaying transition. Because of the increase in lift and decrease in drag, the kite flies faster and produces a higher traction force and power output. During the retraction phase, the relative flow velocity and Reynolds number are lower, and tripping the boundary layer could improve the aerodynamic efficiency. On the pressure side of the airfoil, tripping results in a turbulent instead of a laminar separation of the flow. This has a similarly beneficial effect as the dimples on a golf ball, delaying separation and therefore reducing the drag. Additionally, the lift coefficient is increased, and therefore, the required lift force to keep the system airborne is reached with a lower angle of attack, which increases the safety margin to the stall angle. By tripping the boundary layer on the suction side, the stall angle can be significantly increased during the retraction phase. In this study, the effects from the woven material with surface roughness, from deformation and three‐dimensional flow components are neglected. These are expected to induce perturbations in the boundary layer that trigger the laminar‐turbulent transition of the boundary layer earlier.

## 4 CONCLUSIONS

Previous studies on airfoils with circular leading edge focus only on low Reynolds numbers. However, LEI kites used for AWE systems encounter also high Reynolds numbers. In this paper, a wide range of Reynolds numbers was investigated for a rigid LEI kite airfoil. The effect of boundary layer transition was studied by using the $\gamma -\tilde{R}e_{\theta t}$ transition model and comparing its accuracy to the conventional way of not modeling the laminar boundary layer nor the transition. Both the SST turbulence model and the $\gamma -\tilde{R}e_{\theta t}$ transition model formulations in OpenFOAM v1706 toolbox were presented. The computational setup was verified by carrying out a mesh uncertainty assessment and validated by comparing the simulation results with a low Reynolds number experiment on a similar sailwing airfoil.

For the sailwing airfoil, it was shown that the transition model agrees well with the experimental results over the whole range of Reynolds numbers. By contrast, the results with transition modeling differ significantly from the experimental data at small angles of attack. In particular, the sign of the lift coefficient was positive without the transition model while it was negative in the experiment and in the numerical simulations with the transition model. Moreover, the drag coefficient was underpredicted without transition modeling. For angles of attack larger than 8°, both approaches showed similar results because the small dimple at the junction of leading edge and arc‐shaped plate induces a separation bubble on the suction side of the airfoil. In this range of incidence angles, the boundary layer transitions in the separation bubble, which explains why both models lead to similar turbulent boundary layers behind the bubble. By contrast, for *α* < 8°, the flow does not transition in the separation bubble. Therefore, after reattachment, the laminar boundary layer separates earlier than the turbulent boundary layer without using the transition model.

The simulations on the LEI kite airfoil were performed with a wide range of Reynolds numbers. The results show that it is important to take transition into account for Re < 2 × 10^7^. At low Reynolds numbers, the aerodynamics suffers from laminar separation both on the suction and pressure sides. As the Reynolds number increases, the size of the laminar separation bubble on the suction side decreases, which results in a thinner boundary layer and higher critical angles of attack. For Re > 2 × 10^7^, the transition occurs almost immediately when the flow approaches the airfoil. In this study, the best efficiency is achieved with Re = 5 × 10^6^, at which the flow exhibits the longest laminar boundary layer without laminar separation bubble. Moreover, with low angles of attack on the suction side, the separation occurs from a turbulent boundary layer when Re ≥ 5 × 10^6^, which also improves the aerodynamic efficiency. At large Reynolds numbers, the results obtained without the transition model are in better agreement with those obtained with the transition model. The aerodynamics of the airfoil also improves as the Reynolds number increases due to the decreasing thickness of the turbulent boundary layer.

In this study, we did not account for the effects of the woven membrane material, the typically significant deformations, and the three‐dimensionality of the flow, but recommend to investigate these effects in future studies on the subject.

## References

[1] Bechtle P , Schelbergen M , Schmehl R , Zillmann U , Watson S . Airborne wind energy resource analysis. Renewable Energy. 2018 accepted for publication. Preprint available from https://arxiv.org/abs/1808.07718

[2] Viré A , Schmehl R . How to harness wind energy with traction kites. Rev Environ Sci Biotechnol. 2015;14:1‐4.

[3] Zillmann U , Bechtle P . Emergence and economic dimension of airborne wind energy In: Schmehl R, ed. Airborne Wind Energy – Advances in Technology Development and Research. Singapore:Springer Nature; 2018:1‐25.

[4] Faggiani P , Schmehl R . Design and economics of a pumping kite wind park In: Schmehl R, ed. Airborne Wind Energy – Advances in Technology Development and Research, Green Energy and Technology Singapore:Springer Nature; 2018:391‐411.

[5] Dunker S . Tether and bridle line drag in airborne wind energy applications In: Schmehl R, ed. Airborne Wind Energy – Advances in Technology Development and Research, Green Energy and Technology Singapore:Springer; 2018:29‐56.

[6] Ahrens U , Diehl M , Schmehl R , eds.. Airborne Wind Energy, Green Energy and Technology Berlin Heidelberg:Springer; 2013.

[7] Schmehl R , ed.. The International Airborne Wind Energy Conference 2015: Book of Abstracts. Delft, The Netherlands:Delft University of Technology; 2015.

[8] Diehl M , Leuthold R , Schmehl R , eds.. The International Airborne Wind Energy Conference 2017: Book of Abstracts. Freiburg, Germany:University of Freiburg | Delft University of Technology; 2017.

[9] Schmehl R , ed.. Airborne Wind Energy—Advances In Technology Development And Research, Green Energy and Technology Singapore:Springer Nature; 2018.

[10] van der Vlugt R , Peschel J , Schmehl R . Design and experimental characterization of a pumping kite power system In: Ahrens U, Diehl M, Schmehl R, eds. Airborne Wind Energy, Green Energy and Technology Berlin Heidelberg:Springer; 2013:403‐425.

[11] van der Vlugt R , Bley A , Schmehl R , Noom M . Quasi‐steady model of a pumping kite power system. Renewable Energy. 2019;131:83‐99.

[12] Breukels J , Schmehl R , Ockels W . Aeroelastic simulation of flexible membrane wings based on multibody system dynamics In: Ahrens U, Diehl M, Schmehl R, eds. Airborne Wind Energy, Green Energy and Technology Berlin Heidelberg:Springer; 2013:287‐305.

[13] Bosch A , Schmehl R , Tiso P , Rixen D . Dynamic nonlinear aeroelastic model of a kite for power generation. AIAA J Guidance Control Dyn. 2014;37(5):1426‐1436.

[14] Oehler J , Schmehl R . Aerodynamic characterization of a soft kite by in situ flow measurement. Wind Energy Sci. 2019;4(1):1‐21.

[15] Hummel J , Göhlich D , Schmehl R . Automatic measurement and characterization of the dynamic properties of tethered membrane wings. Wind Energy Sci. 2019;4:41‐55.

[16] Fink M . Full‐scale investigation of the aerodynamic characteristics of a model employing a sailwing concept. Technical Report NASA TN D‐4062, Hampton, VA, USA, NASA Langley Research Center; 1967 http://hdl.handle.net/2027/uiug.30112106870097

[17] Kroo I . Aerodynamics, aeroelasticity, and stability of hang gliders—experimental results. Technical Report NASA‐TM‐81269, Moffett Field, CA, USA, NASA Ames Research Center; 1981 http://hdl.handle.net/2060/19810015490

[18] Maughmer M . Optimization and characteristics of a sailwing windmill rotor. AMS Report, No. 1297. New Jersey: Princeton University; 1976.

[19] Maughmer M . A comparison of the aerodynamic characteristics of eight sailwing airfoil sections. In: Proceedings of the 3rd International Symposium on the Science and Technology of Low Speed and Motorless Flight; 1979; Hampton, VA, USA:155‐176. http://hdl.handle.net/2060/19790015726

[20] Bruining A . Aerodynamic characteristics of a curved plate airfoil section at Reynolds numbers 60.000 and 100.000 and angles of attack from ‐10 to +90 degrees. Report LR‐281, Delft, Netherlands, Delft University of Technology; 1979 http://resolver.tudelft.nl/uuid:6b92442a-01f7-4b7c-8d53-c4f10720ff3e

[21] Boer R . Low speed aerodynamic characteristics of a two‐dimensional sail wing with adjustable slack of the sail. Report LR‐307, Delft, Netherlands, Delft University of Technology; 1980 http://resolver.tudelft.nl/uuid:18ae2cc6-434e-49c8-9296-d3fa450850a5

[22] Bot P , Rabaud M , Thomas G , Lombardi A , Lebret C . Sharp transition in the lift force of a fluid flowing past nonsymmetrical obstacles: evidence for a lift crisis in the drag crisis regime. Phys Rev Lett. 2016;117(23):234501. 10.1103/PhysRevLett.117.234501 27982615

[23] Langtry R , Menter F . Correlation‐based transition modeling for unstructured parallelized computational fluid dynamics codes. AIAA J. 2009;47(12):2894‐2906.

[24] Walters D , Cokljat D . A three‐equation eddy‐viscosity model for Reynolds‐averaged Navier–Stokes simulations of transitional flow. J Fluids Eng. 2008;130(12):1214011‐12140114.

[25] Menter F . Zonal two equation kappa‐omega turbulence models for aerodynamic flows. Proceedings of the 23rd Fluid Dynamics, Plasmadynamics, and Lasers Conference, AIAA Paper 93‐2906; 1993.

[26] OpenFOAM . https://www.openfoam.com/. Accessed: 14 August 2018.

[27] Jones W , Launder B . The prediction of laminarization with a two‐equation model of turbulence. Int J Heat Mass Transfer. 1972;15(2):301‐314.

[28] Wilcox D . Reassessment of the scale‐determining equation for advanced turbulence models. AIAA J. 1988;26(11):1299‐1310.

[29] Menter F , Kuntz M , Langtry R . Ten years of industrial experience with the SST turbulence model In: Hanjalic K., Nagano Y, Tummers M, eds. Turbulence, Heat and Mass Transfer 4. Antalya:Begell House, Inc.; 2003:625‐632.

[30] Menter F , Esch T . Elements of industrial heat transfer prediction. In: Proceedings of the 16th Brazilian Congress of Mechanical Engineering (COBEM); 2001; Uberlandia, Brazil:117‐127.

[31] Rumsey C . Turbulence modeling resource, nasa Langley research center. https://turbmodels.larc.nasa.gov/sst.html. Accessed: 14 August 2018.

[32] Langtry R , Menter F , Likki S , Suzen Y , Huang P , Völker S . A correlation‐based transition model using local variables–part II: test cases and industrial applications. J Turbomachinery. 2006;128(3):423‐434.

[33] Saric WS , Reed HL , White EB . Stability and transition of three‐dimensional boundary layers. Annu Rev Fluid Mech. 2003;35(1):413‐440.

[34] Reshotko E . Boundary‐layer instability, transition and control. In: Proceedings of the 32nd Aerospace Sciences Meeting and Exhibit, AIAA Paper 94‐0001; 1994January; Reno, NV, USA:117‐127.

[35] Medida S , Baeder J . Numerical prediction of static and dynamic stall phenomena using the $\gamma -\tilde{R}e_{\theta t}$ transition model. In: American Helicopter Society 67th Annual Forum; 2011; Virginia Beach, VA, USA: 431‐454.

[36] Zheng Z , Lei J . Application of the *γ* − *re* _*θ*_ transition model to simulations of the flow past a circular cylinder. Flow Turbul Combust. 2016;97(2):401‐426.

[37] Spalart P , Rumsey C . Effective inflow conditions for turbulence models in aerodynamic calculations. AIAA J. 2007;45:2544‐2553.

[38] Brown D , Chesshire G , Henshaw W , Quinlan D . Overture: an object oriented software system for solving partial differential equations in serial and parallel environments. In: Proceedings of the Eighth SIAM Conference on Parallel Processing for Scientific Computing; 1997; Minneapolis: 1‐8.

[39] Walker GJ . The role of laminar‐turbulent transition in gas turbine engines: a discussion. J Turbomach. 1993;115:207‐216.

[40] Achenbach E . Distribution of local pressure and skin friction around a circular cylinder in cross‐flow up to re = 5 × 10^6^ . J Fluid Mech. 1968;34(4):625‐639.

